# Cysts of the Nasal Passages1Read before the Section on Anatomy, Physiology, and Pathology, of the Buffalo Academy of Medicine, September 19, 1893.

**Published:** 1893-10

**Authors:** W. Scott Renner

**Affiliations:** Professor of Laryngology, Medical Department, Niagara University, etc.; 361 Pearl Street


### CYSTS OF THE NASAL PASSAGES.[1]

By W. SCOTT RENNER, M. D.,
Professor of Laryngology, Medical Department, Niagara University, etc.

Until recently, cysts of the nasal passages were scarcely ever mentioned in medical literature, and even since the development of rhinology, and the rapid strides which have been made within the last few years, few cases have been reported.

Four different pathological conditions of the nasal passages have been described under this title. These are glandular retention cysts, cystic degeneration of nasal polypi, or cystic polyp,

1. Read before the Section on Anatomy, Physiology, and Pathology, of the Buffalo Academy of Medicine, September 19, 1893.

osseous cysts, including cysts of the turbinated bones, especially of the middle, and dermoid cysts of the nasal passages.

Before speaking of these different forms of cystic growths, I wish to relate the principal points of a case which occurred in my practice.

The specimen which I now show you is the sac of a multilocular cyst of the nasal passage. It measures about two inches in length and about three-fourths of an inch in breadth. The amount of fluid which it contained, unfortunately, could not be estimated.

I removed this specimen from the left nostril of Mrs. J. N., thirty-five years of age, a charity patient at the Buffalo Eye and Ear Infirmary. She came from the country, and applied for treatment at the Infirmary in October, 1891. She complained of nasal obstruction, headache, and catarrhal symptoms. She stated that only the left side of her nose was constantly obstructed, the other only at times. The left side had troubled her for years. I examined her nose, and found what I supposed was a large nasal polypus filling the left nasal passage, anteriorly. This growth could be easily seen without a speculum by lifting up the tip of the nose. The same or a similar growth could be seen on the same side when making a posterior rhinoscopic examination. It did not, however, extend into the cavity of the naso-pharynx. Although I supposed the tumor was an ordinary soft nasal polypus, it was much more translucent than they generally are. I informed the patient that she had a growth in the left nasal passage, which should be removed in order to relieve her unpleasant symptoms. She consented to the operation, and I operated rapidly with the cold snare. Much to my surprise, as soon as I commenced to encompass the tumor, it burst, and fluid of a light yellow color flowed from the nose. I was compelled to tear the growth from its attachment with some force, when I found that it was a multilocular cyst, of which the largest cavity had been opened when the fluid escaped from the nose, that the growth seen posteriorly was the same one which had presented itself anteriorly, and that the whole growth had been removed. I then applied a cocaine solution thoroughly to the bleeding surface, and was able, in a few minutes, to demonstrate that the growth had had an extensive origin from the free border and anterior end of the middle turbinated bone of the left side. I also discovered that the patient had no other polypoid or other growths in the nose. The patient returned the following day to the country, and has remained well ever since.

I was inclined to think that this cyst was formed from a degenerated nasal polypus. Dr. William C. Krauss examined it, and found it to be a multilocular cyst with no epithelial lining. He considered it a degenerated soft nasal polypus, and not a retention cyst, a condition of the mucous membrane of the nasal passages which has been described by some writers. It is not uncommon to find small cavities, or degenerated spots, in the center of old nasal polypi, as well as in tumors in other localities, and not seldom one of several polypi may have a large cystic cavity in its center, filled with fluid, as was the case with this specimen. I removed this (Specimen No. 2) from the naso-pharynx of a boy, twenty years of age, who came to consult me, from the country, about two years ago. The whole of his nasal and naso-pharyngeal cavities were filled with soft polypi. When I first saw him, the expression of his face was so peculiar that I thought that I had a case of fibroma of the naso-pharynx. The specimen was one of several large polypi which I found extending from their attachment to the middle turbinated bone into the naso-pharynx.

Moure, of Bordeaux, in 1886, reported a case of cystic polyp, partly occluding the posterior nares of the left side, in a patient suffering from mucous polypi of the right side. The growth was removed and expelled through the mouth, the fluid having escaped. This cyst, when distended, was about the size of a small egg. "The author looks upon the growth as a mucous polyp, a portion of which had undergone cystic degeneration, a condition which he does not consider uncommon, especially where polypi pass through the posterior nares into the pharynx."

The late Dr. F. H. Potter, of this city, reported a case of unilocular cyst of the nasal mucous membrane attached to the middle turbinated bone. Besides this, and the case of Moure's, which I have just mentioned, I can find reference to but eleven cases of cysts of the nasal mucous membrane in the literature at my disposal. Park, Johnson, Watson, Bonauno, Zahn, Lefferts, Ingals, C. W. Richardson, and Babcock, have each reported one case, Chatellier two. This list does not, of course, include the reports of osseous and dermoid cysts which may be found in the literature. The original articles I have not been able to consult, in most cases, on account of the short space of time at my command to prepare this paper.

In Watson's case, the tumor was multilocular and attached to the middle turbinated bone. In the cases reported by Lefferts,

Johnson, and Potter, the cysts, although unilocular, were attached to the middle turbinated bones, those of Lefferts and Johnson extending into the naso-pharynx. None of these cases differ materially from Moure's case of degenerated polyp, except in that they were not associated with nasal polypi. These growths, in the four cases enumerated above, resemble Moure's as well as mine, in that they all were attached to the middle turbinated bone, which is the favorite attachment for soft nasal polypi, a fact which would make one suspect that these retention cysts were retention cysts formed in the glandular tissue found in the substance of the so-called myxomatous or soft nasal polypi. Two of these tumors, like Moure's, extended into the naso-pharynx. It is quite common, according to Moure, for polypi extending into the naso-pharynx to undergo cystic degeneration. This statement is supported by the second specimen which I showed you. A third one, Watson's, was, like mine, multilocular and confined to the nasal cavity. It is natural to suppose that a multilocular cyst is much more likely to have its origin in a degenerated polypus than in an obstructed gland. Potter's case was not multilocular, and did not extend into the naso-pharynx. The two reported by Chatellier did not originate from the middle turbinated bones. The point of origin of the other five cases I have been unable to learn.

Whatever the origin of these cysts, whether by distension of a simple gland, or within a degenerating polypus, they are practically the same. In one case they form from the adenoid tissue of the nasal mucous membrane, in the others, probably, from the adenoid tissue found usually in soft nasal polypi.

By the operator, they are usually mistaken for polypi. He, generally, only becomes aware that these growths are cystic after he has commenced operating, except in cases attached far forward in the nose, as they were in both of Chatellier's cases.

Osseous cysts of the nasal passages have been found most frequently in the anterior extremity of the middle turbinated bones. They pass unnoticed until the mucous membrane covering them becomes hypertrophied, when the symptoms of pressure against the septum cause the patient to consult the specialist. All such enlargements of this part of the turbinated bone do not contain a cavity, and no examiner of the nose can be positive of the presence of such a cavity until he has removed the enlarged end of the bone, unless it be of very great size, like the case reported by Knight, of New York.

Tucker Kandl describes these cavities of the middle turbinated bone as an anomalous condition of their development. He does not consider them the result of any pathological process. Some consider them the result of hypertrophic changes. Osseous cysts of the septum and lower turbinated bone have been so described.

Dermoid cysts of the nasal passages are rare and interesting, but foreign to the subject of this paper.

The prognosis in all these cases is good, and the treatment is always surgical.

361 PEARL STREET.

